# Phytochemical profile and rosmarinic acid purification from two Peruvian *Lepechinia Willd.* species (*Salviinae, Mentheae, Lamiaceae*)

**DOI:** 10.1038/s41598-021-86692-3

**Published:** 2021-03-31

**Authors:** Carlos A. Serrano, Gretty K. Villena, Eric F. Rodríguez

**Affiliations:** 1 Laboratorio de Química Orgánica, Universidad Nacional de San Antonio Abad del Cusco, Cusco, Peru; 2 Laboratorio de Micología y Biotecnología, Universidad Nacional Agraria La Molina, Lima, Peru; 3 Herbarium Truxillense (HUT), Universidad Nacional de Trujillo-Perú, Trujillo, Peru

**Keywords:** Plant sciences, Chemistry

## Abstract

The phytochemical profile of *Lepechinia meyenii* (Walp.) Epling and *Lepechina floribunda* (Benth.) Epling obtained by liquid chromatography associated with high-resolution mass spectrometry is presented. Forty eight compounds were detected exhibiting a variety of salvianolic acids and abietane phenolic diterpenoids. A simple procedure by cold evaporative crystallization to purify rosmarinic acid from these botanical species was also shown.

## Introduction

Traditional medicines in Peru like others in different parts of the world are characterized by the use of plants of the big family *Lamiaceae*^1–4^. One of these approaches reports 25 *Lamiaceae* species out of a total of 510 medicinal plants (4.9%)^4^. *Lamiaceae* is a cosmopolitan family with more than 230 genera and approximately 7000 species^5,6^. The *Lamiaceae* family in Peru has about 21 genera and 190 species, mostly herbs and shrubs, 57 species are endemic in 9 genera^7^. *Lamiaceae* has twelve subfamilies, one of them being *Nepetoideae* (3400 species, 105 genera)^6,8^. *Mentheae* is the largest and economically important tribe of the *Nepetoideae* subfamily (2000 species, 60 genera, 3 tribes)^6,9^. The *Mentheae* tribe in turn is divided into five sub-tribes: *Menthinae, Salviinae, Nepetinae, Prunellinae* and *Lycopinae*^6,9,10^. In Peru *Salviinae* tribe is represented fundamentally by two genera: *Lepechinia and Salvia*. The pan american genus *Lepechinia* Willd. is constituted by ca 45 species, 30 of which occur in South America at elevations from 1500 to 4000 m within a broad range of habitats^11,12^. *Lepechinia* in Peru has 4 endemic species: *Lepechinia marica* Epling & Mathias*, Lepechinia mollis* Epling*, Lepechinia scobina* Epling *Lepechinia tomentosa* (Benth.) Epling ^6^. In the Mentheae tribe, the presence of volatile and non-volatile terpenoids, the absence of iridoids (monoterpenglycosides) and the abundance of rosmarinic acid and their higher derivatives, salvianolic acids, is characteristic^13^. The presence of rosmarinic acid is not exclusive to *Mentheae*, but for all *Nepetoideae*^6,13^. In contrast, rosmarinic acid is not present in the *Lamoideae* subfamily, but iridoids are^13^. In addition, it is known that rosmarinic acid is also present in very diverse taxa in dicots, monocots, ferns and hornworts and that for this reason it is not a good chemotaxonomic indicator but very useful to distinguish intrafamilial taxonomic subgroups in *Lamiaceae*^14^. This varied taxonomic distribution suggests a complicated evolutionary history in rosmarinic acid biosynthesis, for example *Lamiaceae* and *Boraginaceae* (order *Lamiales*) have different mechanisms for producing rosmarinic acid^15^ so it is likely that each case has different biosynthetic mechanisms. Rosmarinic acid and salvianolic acids, particularly salvianolic acid A and B has interesting effects on fibrosis, cancer and neurodegenerative processes^16–18^. Other important effects of rosmarinic acid are as an anti-inflammatory, UV protector, antioxidant, cytoprotector^18^ and as an antihepatotoxic^19^. *Lepechinia meyenii* (Walp.) Epling is a medicinal herb that grows at 3800–4000 m of altitude where is employed for respiratory diseases^2^. In the southern andes of Perú this plant “Puna Salvia”^20^ was the third most used plant in communities whose health then depended almost exclusively on the use of medicinal plants. *Lepechinia floribunda* (Benth.) Epling is a sub-shrub that grows in the foothills of the eastern Andes of southern Peru and according to the inhabitants of the collection area is used as a tonic and comforting for the body. In a recent work on Argentinean *Lepechinia meyenii* (Walp.) Epling^21,22^ was reported the presence of three hydroxycinnamic acids: caffeic, p-coumaric and rosmarinic acids and seven abietane diterpenoids: carnosol, rosmanol, carnosic acid, carnosic acid ɤ-lactone, 20-methyl carnosate, 11,12-*O*-diacetylmethyl carnosate and 11,12-*O*-diacetylcarnosic acid, showing antityrosinase and antibacterial properties. The published works with *L. floribunda* (Benth.) Epling deal with the essential oil obtained from plants of Argentina and Bolivia^23,24^. In a previous work we reported, for both species, *L. meyenii* and *L. floribunda*, the total phenolics content (50.00 and 20.77 µg gallic acid/100 µg ethanolic extract), antioxidant activity (25.79 and 14.11 µg ascorbic acid/100 µg ethanolic extract) and the rosmarinic acid content (4.61 and 1.43%)^25^. The rosmarinic acid content in these two Lepechinias is high when compared with the content in species of the genus Salvia from other parts of the world^26,27^. *Lepechinia* also has a higher rosmarinic acid content than Peruvian *Salvia*, and than Peruvian *Minthostachys*, *Clinopodium* and *Hedeoma* (*Menthinae*)^25^.

Considering all the studies mentioned above, we see that *Lepechinia meyenii* (Walp.) Epling and *Lepechinia floribunda* (Benth.) Epling have not been fully investigated for their non-volatile composition. Ultra-performance liquid chromatography associated with tandem mass spectrometry (UHPLC/MSMS) is an important structural tool for the study of complex plant extracts, for this we will use the UHPLC-Q-OT-MS technology, the Q-Exactive mass spectrometer hybridizes the high mass resolving power of orbitrap mass analyzer with the selectivity of a quadrupole, multiple precursor ions are fragmented in a high energy collision cell and the product ions could be detected with a mass error of less than 5 ppm for a wide range of analyte concentrations^28,29^. Data processing includes spectral similarity prospection and characteristic product ions—neutral loss searching^29,30^.

In the present work, the first objective is the phytochemical profile of the ethanolic extract of the aerial parts of both plants by UHPL-Q-OT-MS. And, our second objective is the purification of rosmarinic acid from these two species.

## Results

### Phytochemical profile

**Figure 1:**
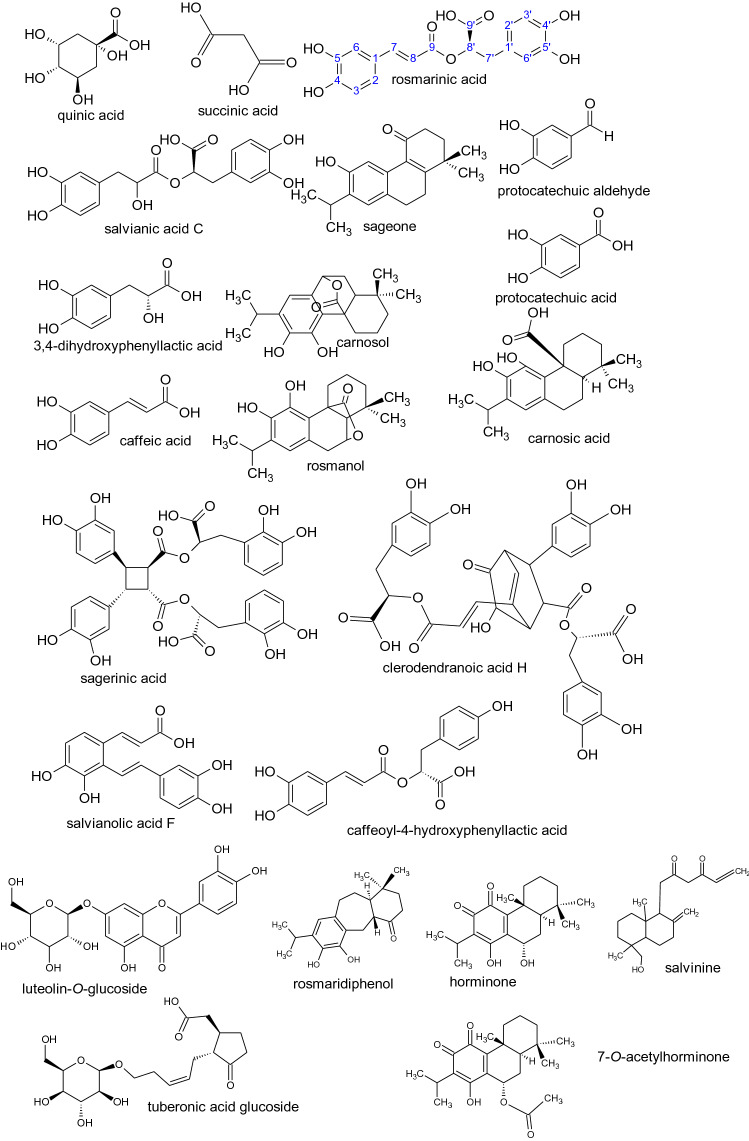
Detected compounds in *Lepechinia meyenii* (Walp.) Epling and *Lepechinia floribunda* (Benth.) Epling.

**Figure 2:**
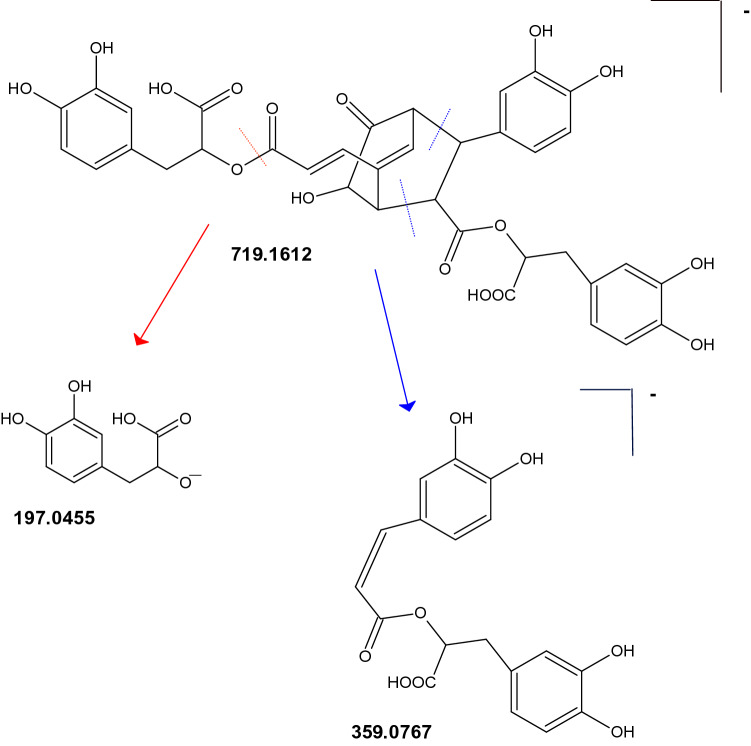
Proposed fragmentation of clerodendranoic acid H.

**Figure 3:**
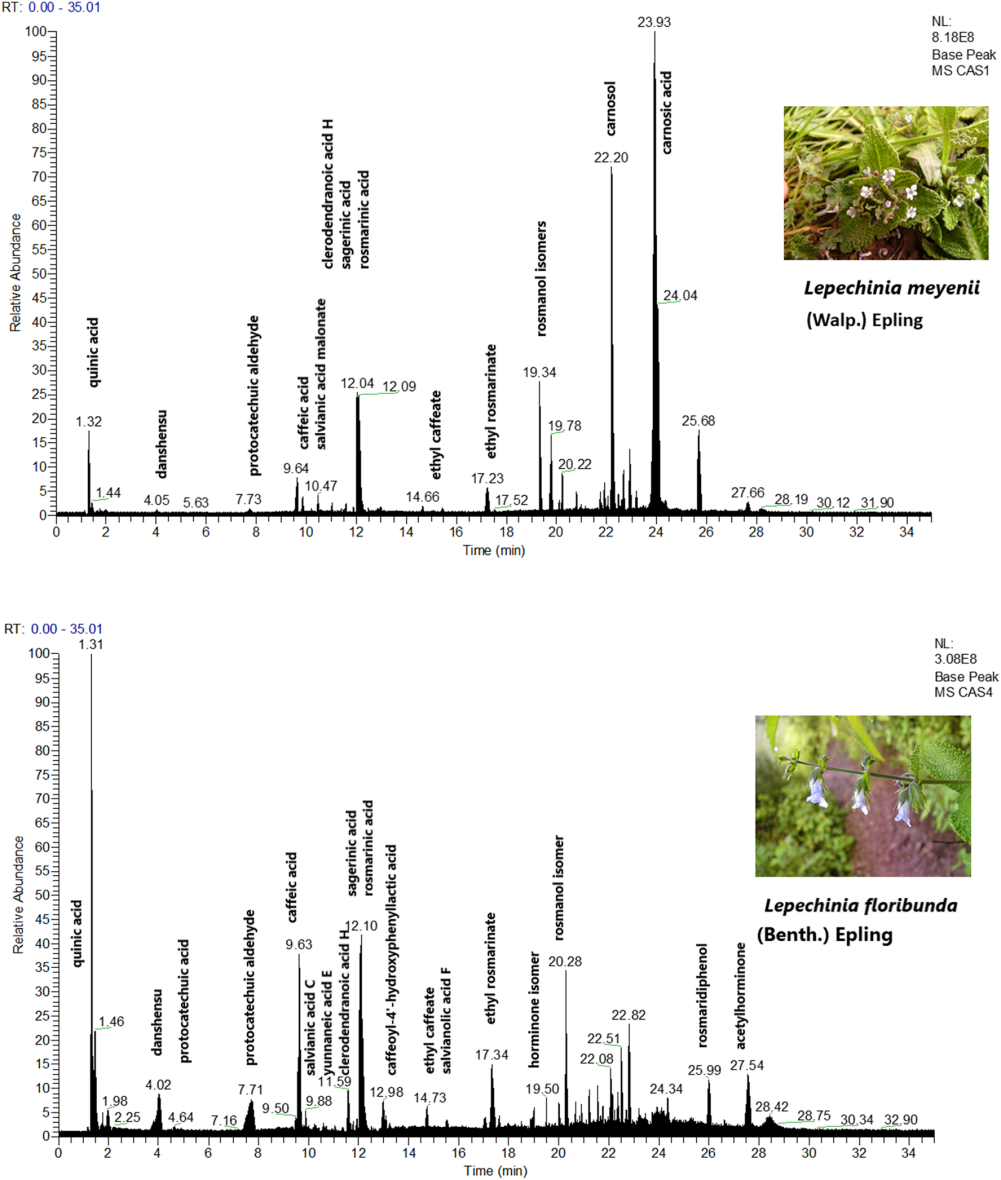
UHPLC/MSMS chromatograms of ethanolic extracts of *L. meyenii* and *L. floribunda.*

The phytochemical profile of the ethanolic extract of *Lepechinia meyenii* (Walp.) Epling and *Lepechinia floribunda* (Benth.) Epling were obtained in negative mode and the detected compounds appears in Table 1. The structures are shown in the Fig. 1. Assignments were made based on data published in the literature^30–41^. We found the free monomers 3,4-dihydroxyphenyllactic acid “danshensu” (*m/z* 197.0450)^30,33^, caffeic acid (*m/z* 179.0345) , protocatechuic aldehyde *(m/z* 137.0239) and protocatechuic acid (*m/z* 153.0188). Fragments *m/z* 197.0450 of danshensu and *m/z* 179.0345 of caffeic acid appear in the mass spectra of all salvianolic acids, they are diagnostic ions in the ion filtering strategy^30^. Dimeric salvianolic acids were rosmarinic acid (*m/z* 359.0767)^34,35^, salvianic acid C (*m/z* 377.0873)^31^, (caffeoyl-4-hydroxyphenyl)lactic acid, “isorinic acid”^60^ (*m/z* 343.0818)^31^, and salvianolic acid F (*m/z* 313.0712)^31^. Salvianic acid C is a molecule that results from hydration of rosmarinic acid of which there is little information. The fragment *m/z* 359.0767 of rosmarinic acid it is also a diagnostic ion for the larger salvianolic acids. A trimeric salvianolic acid were yunnaneic acid E (*m/z* 571.1088)^33^. Tetrameric salvianolic acids were sagerinic acid (*m/z* 719.1612)^34,35^ and clerodendranoic acid H *(m/z* 719.1612)^33,40^, Fig. 2. In both *Lepechinias* we found the phenolic diterpenoids carnosol (*m/z* 329.1752) [M-H-CO_2_]^−^^36,37^, rosmanol (*m/z* 345.1702) [M-H-CO_2_-H_2_O]^−^^36,37^, carnosic acid (*m/z* 331.1910) [M-H-CO_2_-iPr]^−^^36,37^. The diagnostic ions are *m/z* 285.1861 for carnosol, *m/z* 283.0616 for rosmanol and *m/z* 287.2017 for carnosic acid^37^. Other diterpenoid structures are rosmaridiphenol (*m/z* 315.1960)^39,41^, sageone (*m/z* 299.1647), the diterpenoid dicetone salvinine (*m/z* 317.2117), and the phenantrenequinone horminone (*m/z* 331.1910)^38^. The structure presented for rosmaridiphenol has the carbonyl in position 1 and not in position 20 as established in^41^. Also, as can be seen, several of the assignments correspond to minor modifications of the structures described: Ethyl rosmarinate (*m/z* 387.1080)^30^ , Methyl rosmarinate (*m/z* 373.0924)^30^, ethyl caffeate *(m/z* 207.0657)^30^, vinyl caffeate (*m/z* 205.0501), carnosol, rosmanol and carnosic acid derivatives and acetylhorminone. Vinyl caffeate is not a rarity, it has been isolated from plants of the *Perilla* and *Isodon* genus (*Lamiaceae*)^42,43^, has described methods to synthesize it^44,45^ and serves as a precursor to synthesize chlorogenic acids^46^. Also, note the presence of quinic acid (*m/z* 191.0556) but with the absence of chlorogenic acids. The almost null presentation of flavonoids, only luteolin-O-hexoside (m/z 447.0928) in *Lepechinia meyenii* (Walp.) Epling and the presence of the glucoside of tuberonic acid (*m/z* 387.1655) which is a growth hormone. The HPLC / MSMS chromatograms of both *L. meyenii* and *L. floribunda* are shown.in Fig. 3.

**Table 1:** Compounds detected in the ethanolic extract of *Lepechinia meyenii* Walp. (Epling) *(Lm)* and *Lepechinia floribunda* (Benth.) Epling *(Lf)* by UHPLC/MSMS.

Peak	Assignment	*Lm*	*Lf*	tR (min.)	[M-H]^−^	Theoretical mass (*m/z*)	Experimental mass (*m/z*)	Error (ppm)	Ions (*m/z*)
1	Quinic acid	+	+	1.32	C_7_H_11_O_16_	191.0556	191.0559	1.57	127.0395
2	Quinic acid isomer	+	+	1.46	C_7_H_11_O_16_	191.0556	191.056	2.09	127.8696
3	Succinic acid	−	+	1.98	C_4_H_5_O_4_	117.0188	117.0188	0	
4	3,4-dihydroxyphenyl lactic acid “danshensu”	+	+	4.02	C_9_H_9_O_5_	197.045	197.0454	2.03	135.0447, 179.0347^30,33^
5	protocatechuic acid	−	+	4.64	C_7_H_5_O_4_	153.0188	153.019	1.31	135.0448, 109.0289
6	Protocatechuic aldehyde	+	+	7.71	C_7_H_5_O_3_	137.0239	137.024	0.73	108.0209, 119.0341
7	caffeic acid	+	+	9.63	C_9_H_7_O_4_	179.0345	179.0348	1.68	135.0447
8	Tuberonic acid glucoside	+	+	9.88	C_18_H_27_O_9_	387.1655	387.1666	2.84	163.0034, 207.0296, 101.0236
9	Salvianic acid C or isomer	+	+	10.48	C_18_H_17_O_9_	377.0873	377.0884	2.92	359.0777, 197.0454, 347.0776, 137.0240^31^
10	Yunnaneic acid E	−	+	10.54	C_27_H_23_O_14_	571.1088	571.1093	0.88	391.0674, 373.0576, 347.0784, 285.0773, 197.0455, 179.0357, 161.0241,135.0447; 527.1197[M-carboxyl]^−^,329.0672[M-H-carboxyl-danshensu]^−^^33^
11	Luteolin-O-hexoside	+	−	11.02	C_21_H_19_O_11_	447.0928	447.0936	1.78	285.0406
12	salvianic acid C malonate	+	+	11.05	C_21_H_19_O_12_	463.0877	463.0887	2.15	267.0662, 377.1822, 359.0776
13	Clerodendranoic acid H	+	+	11.59	C_36_H_31_O_16_	719.1612	719.1602	1.39	359.0779,179.0347, 197.0452, 161.0241, 135.0447, 539.1193; 522.1127[M-danshensu-H]^-^, 629.1240[M-2carboxyl-H]^−^^33,40^
14	Sagerinic acid	+	+	11.93	C_36_H_31_O_16_	719.1612	719.1601	1.53	161.0241, 179.0347, 359.0775, 539.1188^34,35^
15	Rosmarinic acid	+	+	12.1	C_18_H_15_O_8_	359.0767	359.0776	2.51	161.0240, 179.0346, 197.0453^34,35^
16	(caffeoyl-4′-hydroxyphenyl)lactic acid, “isorinic acid”	+	+	12.98	C_18_H_15_O_7_	343.0818	343.0829	3.21	161.0241, 327.2182^31^
17	methylrosmarinate	−	+	13.28	C_19_H_17_O_8_	373.0924	373.0935	2.95	359.0778, 194.0540, 179.0347
18	ethyl caffeate	+	+	14.73	C_11_H_11_O_4_	207.0657	207.0662	2.4	179.0346
19	salvianolic acid F	+	+	15.53	C_17_H_13_O_6_	313.0712	313.0721	2.88	269.0822[M-carboxyl]^−^, 159.0657^31^
20	vinyl caffeate	−	+	17.05	C_11_H_9_O_4_	205.0501	205.0505	1.95	162.0193 [Caffeoyl-H]^−^
21	Ethyl rosmarinate	+	+	17.34	C_20_H_19_O_8_	387.108	387.1089	2.6	359.0776, 206.9724, 179.0346^30^
22	salvianolic acid F isomer	−	+	17.58	C_17_H_13_O_6_	313.0712	313.0721	2.88	269.0821, 159.0448^31^
23	hydroxycarnosic acid	−	+	18.5	C_20_H_27_O_5_	347.1859	347.1869	2.88	303.1606[M-H-CO_2_]^−^, 331.1918 [M-OH]^−^
24	hydroxycarnosic acid isomer	−	+	18.92	C_20_H_27_O_5_	347.1859	347.1869	2.88	303.1234[M-H-CO_2_]^−^, 331.1919 [M-OH]^−^
25	Horminone or isomer	−	+	19.5	C_20_H_27_O_4_	331.191	331.1919	0.3	112.9850, 170.8329, 197.5107, 301.1813, 313.0712, 456.0566, 492.0332^38^
26	Horminone or isomer	−	+	20.05	C_20_H_27_O4	331.191	331.1919	0.3	112.9850, 170.8328, 197.5106, 313.2389, 456.0567, 492.0332^38^
27	Rosmanol isomer	+	+	20.28	C_20_H_25_O_5_	345.1702	345.1712	2.9	283.0616^36,37^
28	hydroxycarnosic acid isomer	+	−	20.52	C_20_H_27_O_5_	347.1859	347.1866	2.02	331.1918 [M-OH]-
29	Rosmanol isomer	+	−	20.79	C_20_H_25_O_5_	345.1702	345.1710	2.32	283.1707^36,37^
30	oxorosmanol	+	−	20.94	C_20_H_23_O_6_	359.1495	359.1503	1.39	315.1607 [M-H-CO_2_]^−^
31	Oxorosmanol isomer	+	−	21.21	C_20_H_23_O_6_	359.1495	359.1503	1.39	315.1606 [M-H-CO_2_]^−^
32	hydroxyrosmanol	+	−	21.44	C_20_H_25_O_6_	361.1651	361.1659	2.22	317.1760[M-H-CO_2_]^−^
33	dehydrorosmanol	+	−	21.76	C_20_H_23_O_5_	343.1546	343.1553	2.04	299.1650[M-H-CO_2_]^−^
34	sageone	+	−	21.91	C_19_H_23_O_3_	299.1647	299.1652	1.67	256.1107[M-H-methyl]^−^
35	methylrosmanol	+	−	22.03	C_21_H_27_O_5_	359.1859	359.1867	2.23	345.1711[M-H-isopropyl]^−^
36	hydroxycarnosic acid isomer	−	+	22.14	C_20_H_27_O_5_	347.1859	347.1869	2.88	331.1919 [M-OH]^−^
37	carnosol	+	−	22.2	C_20_H_25_O_4_	329.1752	329.1761	2.70	285.1861[M-H-CO_2_]^−^^36,37^
38	isocarnosol	+	−	22.46	C_20_H_25_O_4_	329.1752	329.1760	2.43	285.1861[M-H-CO_2_]^−^^36,37^
39	Dehydrorosmanol isomer	+	−	22.66	C_20_H_23_O_5_	343.1546	343.1554	2.33	299.1653[M-H-CO_2_]^−^
40	ethylrosmanol	+	−	22.93	C_22_H_29_O_5_	373.2014	373.2022	2.14	329.1761[M-H-CO_2_]^−^
41	ethyl hydroxycarnosate	−	+	23.48	C_22_H_31_O_5_	375.2172	375.2182	2.67	347.1869 [M-H-ethyl]^−^
42	carnosic acid	+	+	23.93	C_20_H_27_O_4_	331.191	331.1917	2.11	287.2017^36,37^
43	rosmaridiphenol	−	+	25.98	C_20_H_27_O_3_	315.196	315.1969	2.86	285.1853[M-H-2methyl]^−^^39,41^
44	ethyl carnosate	−	+	26.62	C_22_H_31_O_4_	359.2223	359.2232	2.51	331.1919 [M-ethyl]^−^
45	Acetylhorminone isomer	+	−	24.04	C_22_H_29_O_5_	373.2015	373.2022	1.88	331.1916 [M-acetyl]^−^
46	Salvinine or isomer	+	−	25.68	C_20_H_29_O_3_	317.2117	317.2125	2.52	287.2012[M-hydroxymethyl]^−^
47	Salvinine or isomer	+	−	27.68	C_20_H_29_O_3_	317.2117	317.2124	2.21	287.2013[M-hydroxymethyl]^−^
48	Acetylhorminone isomer	−	+	28.41	C_22_H_29_O_5_	373.2015	373.2024	2.41	331.1918 [M-acetyl]^−^

### Rosmarinic acid purification

**Figure 4:**
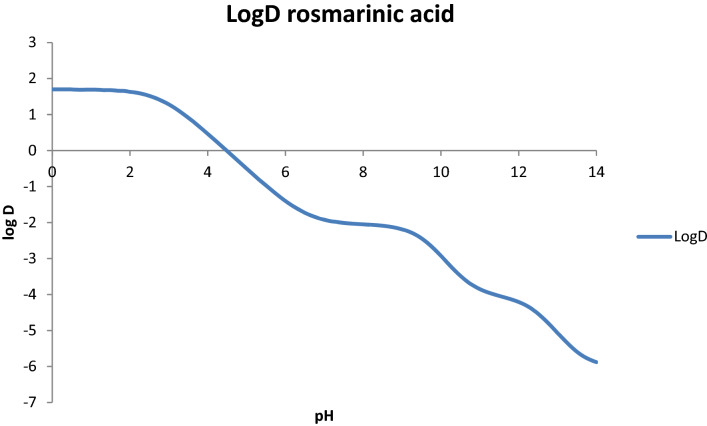
ACD Labs simulated Log D of rosmarinic acid.

The purification of rosmarinic acid was carried out based on the methodology described in^47,48^ using as initial extractant 50% ethanol instead of pure ethanol^49–52^. After evaporating the alcohol from the extract, purification involves adjusting the pH to 2–2.5 and successive partitions with ethyl acetate as described in **3.6**. The choice of this pH value is obtained by simulating the log D of the rosmarinic acid molecule (Fig. 4). Log D are the logP values but over the entire pH range, 0–14.

The final stage of purification involves precipitating solid rosmarinic acid from a concentrated aqueous solution. This was done by placing said solution in a vacuum desiccator with a strong desiccant such as sulfuric acid, which concentrates the cold solution. The solid obtained can be recrystallized by the same procedure.

**Figure 5:**
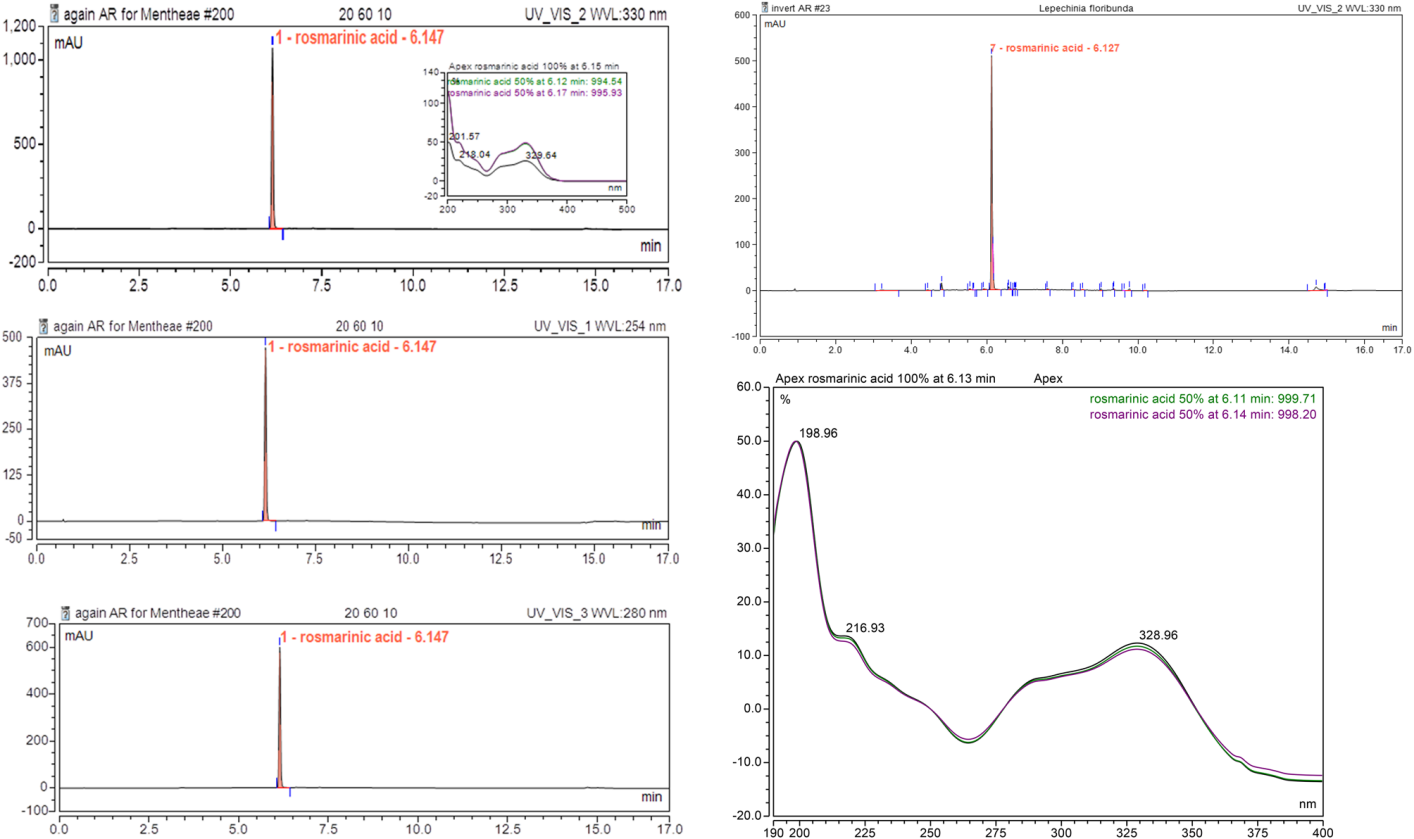
Chromatographic purity of rosmarinic acid at three different wavelengths.

The yields of rosmarinic acid were 2.50% for *L. meyenii* and 1.01% for *L. floribunda* with an analytical PDA-UHPLC purity > 98% (330, 254 and 280 nm), see Fig. 5 and 3.8. The beige solid was characterized with UV, ^1^H-NMR and ^13^C-NMR techniques.

## Discussion

This is the first UHPLC/MSMS phytochemical profile of *L. meyenii* and *L. floribunda* showing salvianolic acids and diterpenoids like principal components. There are the typical monomers, caffeic acid, protocatechuic aldehyde , protocatechuic acid and “danshensu”, which are considered to be the building blocks of dimeric salvianolic acids, rosmarinic acid, salvianic acid C , (caffeoyl-4-hydroxy-phenyl)lactic acid “isorinic acid” and Salvianolic acid F. The trimeric Yunnaneic acid E, salvianolic acid F and the isomeric tetramers sagerinic and clerodendranoic acid H are the more structured salvianolic acids. In addition to the salvianolic acids, we found the diterpenoid phenolics rosmanol, carnosol, carnosic acid and rosmaridiphenol, beside the phenantrenequinone horminone, the same terpenoids as *Rosmarinus officinalis*, an old world medicinal *Mentheae-Salviinae*. These substances support the presence of *Lepechinia* within *Salviinae* beside *Salvia, Melissa, Rosmarinus*, among other genera^27,31,32,34,36,39,54,56,57,59^. However, a recent work^53^, based on DNA, chloroplastic, nuclear ribosomal and low-copy nuclear gene regions, lumps the small genera *Dorystaechas, Meriandra, Perovskia, Rosmarinus* and *Zhumeria* within *Salvia* genus while *Melissa* and *Lepechinia* do not. It is also observed that in the analyzed *Lepechinias*, flavonoids and chlorogenic acids are not significantly present as in the case of *Rosmarinus*^54^. In this work, a luteolin hexoside has been detected in *Lepechinia meyenii* as the only flavonoid in the same way as, luteolin-3-*O*-glucuronide, is shown in *Melissa officinalis*^34,58^.

The rosmarinic acid of both species were easily purified from the hydroethanolic extract without any preparative chromatographic method by a classical procedure^47^ with an initial extraction which adapts the concept that extractability is not the same as solubility—rosmarinic acid is much more soluble in ethanol than in water, but hydroalcoholic mixtures access vacuoles more effectively than pure ethanol because it makes non-permeable to vacuolar membrane^49,55^. Then, partitions with low toxicity solvent and precipitation of rosmarinic acid by cold evaporation of the aqueous solution with the help of a desiccant *in vacuo* and final recrystallization from hot aqueous solution. The yields of rosmarinic acid are approximately half of those of the analytical report^25^. If the solution is simply left to the environment, it takes too long or nucleation conditions are never reached. This process of evaporating aqueous solutions without the application of heat could be applied to purify other types of phenolic acids that are usually difficult to precipitate and tend to remain glassy. Thus, high purity rosmarinic acid has been prepared that can be used as a chromatographic standard to study other botanical species and in the characterization of natural medicines.

## Methods

### Plant material

*Lepechinia meyenii* (Walp.) Epling was collected at the archaeological site of Tambomachay (− 13°28′; − 71°,58′; altitude 3800 m) and *Lepechinia floribunda* (Benth.) Epling was collected at the Urubamba Valley (− 13°31′; − 72°, 06′; altitude 3160 m) in Cusco-Perú. The material was collected by Carlos A. Serrano .Voucher specimen was deposited at Herbarium Truxillense of Universidad Nacional de Trujillo –Perú (HUT 59,504 and 59,503) and identified by the botanist Eric Frank Rodríguez.

### Sample preparation for metabolite fingerprinting

50 mg of powdered aerial parts were subjected to ultrasonic bath for 5 min with 1 mL of ethanol × 3 times. The filtrates were dried *in vacuo* and stored at 4 °C until use.

### UHPLC-Q-OT-MS ^28^

A Thermo Scientifc Dionex Ultimate 3000 UHPLC system equipped with a quaternary Series RS pump and a Thermo Scientifc Dionex Ultimate 3000 Series TCC-3000RS column compartments with a Thermo Fisher Scientifc Ultimate 3000 Series WPS-3000RS autosampler and a rapid separations PDA detector controlled by Chromeleon 7.2 Software hyphenated with a Thermo high resolution Q Exactive focus mass spectrometer were used for analysis. The chromatographic system was coupled to the MS with a Heated Electrospray Ionization Source II (HESI II). Nitrogen (purity > 99.999%) obtained from a Genius NM32LA nitrogen generator was employed as both the collision and damping gas. XCalibur 2.3 software and Trace Finder 3.2 were used for UHPLC control and data processing, respectively. Q Exactive 2.0 SP 2 was used to control the mass spectrometer.

### LC parameters^28^

An UHPLC C18 column (Acclaim, 150 mm × 4.6 mm ID, 5 μm, Thermo Fisher Scientific operated at 25 °C was employed. The detection wavelengths were 255, 280, 355 and 640 nm. PDA was recorded from 200 to 700 nm, and mobile phases were 0.1% formic aqueous solution (A) and acetonitrile (B). The gradient program [time (min), % B] was: (0.00, 5); (5.00, 5); (10.00, 30); (15.00, 30); (20.00, 70); (25.00, 70); (35.00, 5) and 12 min for column equilibration before each injection. The flow rate was 1.0 mL min − 1 , and the injection volume was 10 μL. Plant extracts dissolved in 1.5 mL of methanol , filtered with a 0.22 μm Teflon membrane and were kept at 10 ◦ C inside the autosampler.

### MS parameters^28^

The HESI (Heated Electrospray Ionization Probe) has a sheath gas flow rate of 75 units; the auxiliary gas flow of 20 units; capillary temperature 400° C; auxiliary gas heater temperature 500° C; spray voltage of 2500 V (ESI -). Scanning range of 100/1500 m/ z; scan speed 1 scan / s; 40 eV collision energy; resolution 35,000; negative polarity. The detection was based on the exact mass calculation. The mass tolerance threshold was 5 ppm. Data acquisition and processing were carried out using XCalibur Version 2.3 (Thermo Fisher Scientific).

### Purification of rosmarinic acid

50 g of pulverized aerial parts of *L.meyenii*./*L.f*loribunda was extracted with 500 mL of 50% (v/v) ethanol per ten minutes in the ultrasonic bath at 60 °C per three times. The collected filtrates were evaporated to eliminate the ethanol. The aqueous solution was brought to pH 2.3 and partitioned with ethyl acetate. The ethyl acetate extract was evaporated to dryness and redissolved in hot water. The aqueous solution at 4 °C per 12 h precipitates resinous material. The clear supernatant liquid was again partitioned with ethyl acetate, evaporated to dryness and dissolved in minimal volume of hot water. This aqueous solution in a vaccum desiccator with fresh sulfuric acid precipitates the rosmarinic acid. The product was recrystallized from hot water to produce 1250 or 503 mg of a beige solid, respectively. The product was characterized by HPLC and NMR methods.

### Spectrometric Identification of Rosmarinic acid

Bruker ARX 400; ^1^H-NMR (in CD_3_-CO-CD_3_, 400 MHz): δ 3.04 (2H, m, H-7′), 5.24 (1H, m, H-8′), 6.32(1H, d, J = 15.9 Hz, H-8), 6.70 (1H, dd, *J* = 8.1, 2.1, H-6′), 6.77 (1H, d, *J* = 8, H-5′), 6.87 (1H, d, *J* = 2.0, H-2′), 6.89 (1H, d, *J* = 8.2, H-5), 7.08 (1H, dd, *J* = 8.2, 2.1 Hz, H-6), 7.19 (1H, d, *J* = 2.1 Hz, H-2), 7.29 (1H, d, *J* = 15.9 Hz, H-7). ^13^C-NMR (in CD_3_-CO-CD_3_, 101 MHz): δ 171.16 (C-9′), 166.83 (C-9), 148.95 (C-4), 146.59 (C-3), 146.33 (C-7), 145.75 (C-3′), 144.84 (C-4′), 129.19 (C-1′), 127.51 (C-1), 122.78 (C-6), 121.69 (C-6′), 117.36 (C-2′), 116.41(C-5), 116.00 (C-5′), 115.32(C-2), 114.95(C-8), 73.73(C-8′), 37.49(C-7′). The data were compared with^56^. Spectra of rosmarinic acid appear in supplementary material.

### Analytical PDA-UHPLC

(Dionex Thermoscientific Ultimate 3000 UHPLC with Chromeleon 7.2 software): 100 × 2.1 mm × 1.8 µm Zorbax Rapid Resolution RPC18 column. Separation temperature: 40° C. Flow: 0.4 mL / minute. Gradient: a) H_2_CO_2_ 0.1%; b) MeCN; (time,% b)): (0.0); (1.0); (6.40); (9,100); (13,100); (14.0); (17.0). DAD: 200–500 nm; UVVis 1: 254 nm; UVVis 2: 330 nm; UV Vis 3: 280 nm; UV Vis 4: 370 nm.

## Disclosure statement

The authors declare no conflict of interest. C.S. declares that the botanical material collected was made with permission of Universidad Nacional de San Antonio Abad del Cusco-PERU in quantities less than 300 g of dried material in accord to institutional rules.

## Supplementary Information

Supplementary Information
