# Supplementary material for: Phytochemical profile and rosmarinic acid purification from two Peruvian Lepechinia Willd. species (Salviinae, Mentheae, Lamiaceae)
Source: Sci Rep. 2021 Mar 31;11:7260. doi: 10.1038/s41598-021-86692-3 (PMC8012630; doi:10.1038/s41598-021-86692-3)
Supplement: Supplementary file 1 — Supplementary Information [file 41598_2021_86692_MOESM1_ESM.docx]

**SUPPLEMENTARY MATERIAL:**

**Phytochemical profile and rosmarinic acid purification from two Peruvian *Lepechinia Willd.* species (*Salviinae, Mentheae, Lamiaceae*)**

**Carlos A. Serrano^a^, Gretty K. Villena^b^, Eric F. Rodriguez^c^**

**^a^Laboratorio de Química Orgánica, Universidad Nacional de San Antonio Abad del Cusco, Perú. carlos.serrano@unsaac.edu.pe.**

**^b^Laboratorio de Micología y Biotecnología, Universidad Nacional Agraria La Molina, Lima-Perú.**

**^c^ Herbarium Truxillense (HUT), Universidad Nacional de Trujillo-Perú.**

**Abstract:** The phytochemical profile of *Lepechinia meyenii* (Walp.) Epling and *Lepechina floribunda* (Benth.) Epling obtained by liquid chromatography associated with high-resolution mass spectrometry is presented. Forty eight compounds were detected exhibiting a variety of salvianolic acids and abietane phenolic diterpenoids. A simple procedure by cold evaporative crystallization to purify rosmarinic acid from these botanical species was also shown.

**Keywords:** *Lepechinia meyenii* (Walp.) Epling*,* *Lepechina floribunda* (Benth.) Epling *,Salviinae, Mentheae, Lamiaceae*, UHPLC-Q-OT-MS, salvianolic acids, diterpenoids, rosmarinic acid, cold evaporative crystallization.

**CONTENT**

**S1. ^1^HNMR spectrum of rosmarinic acid (Lf)**

**S2. ^13^CNMR spectrum of rosmarinic acid (Lf)**

**S3. References of supplementary material**

**S1. ^1^HNMR spectrum of rosmarinic acid (400 MHz, acetone d_6_)**


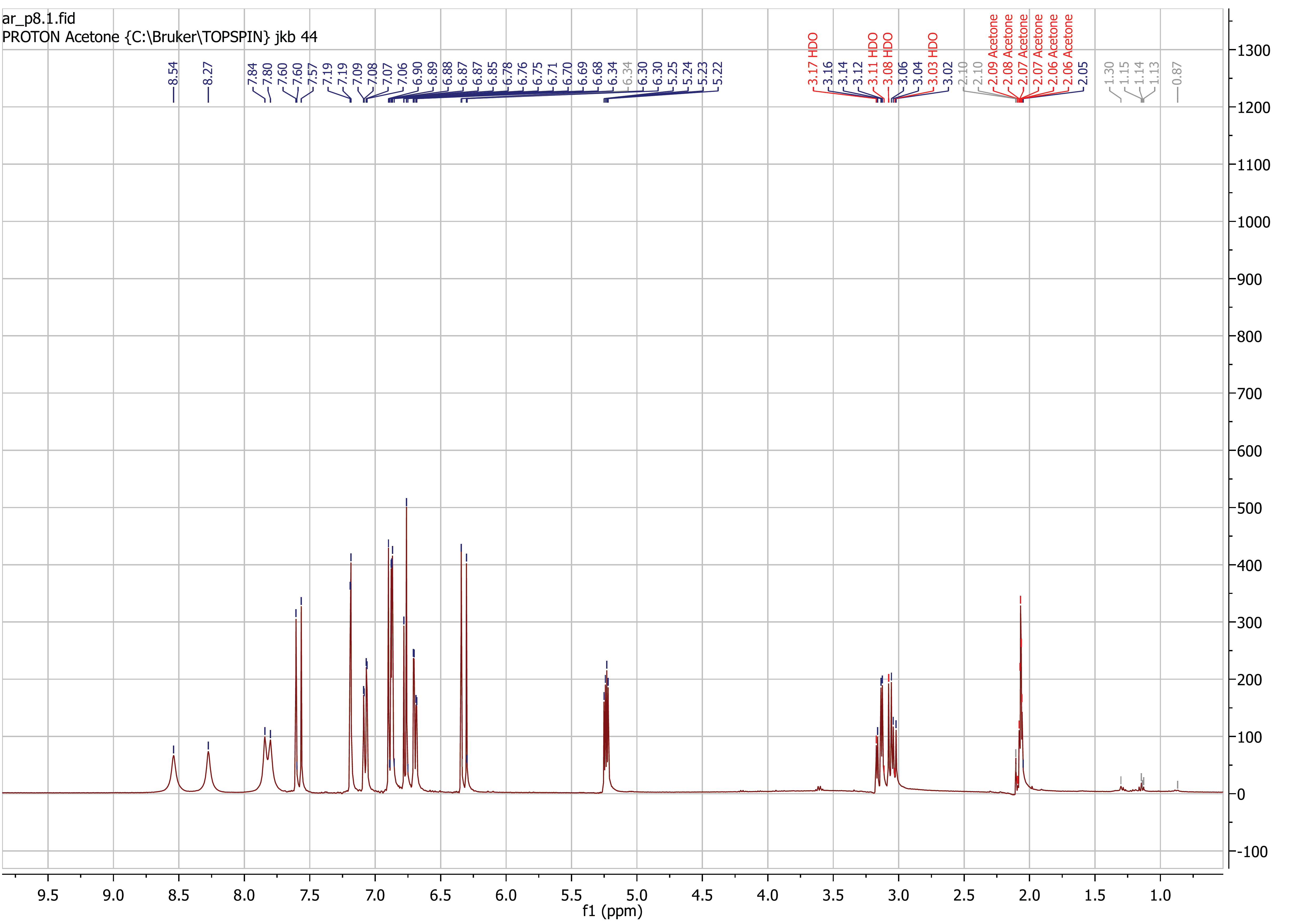

| Wang et al. [**43**] | H |
| --- | --- |
| 3.03, 2H, m | 7’ |
| 5.20, 1H, m | 8’ |
| 6.29, 1H,d, *J* = 16 Hz | 8 |
| 6.61-6,87, 4 H,m | 5, 2’, 5’, 6’ |
| 7.03, 1H, dd, *J* = 8.0, 2.0 Hz | 6 |
| 7.16, 1H, d, *J* =2.0 Hz | 2 |
| 7.55, 1H, d, *J* = 16.0 Hz | 7 |

| δ(ppm), hydrogens, multiplicity | H |
| --- | --- |
| 3.04, 2 H, m | 7’ |
| 5.24, 1 H, dd, *J* = 8.5, 4.2 Hz | 8’ |
| 6.32, 1H, d, *J* = 15.9 Hz | 8 |
| 6.70, 1 H, dd, *J* = 8.1, 2.1 | 6’ |
| 6.77, 1 H, d, *J* = 8 | 5’ |
| 6.87, 1 H, d, *J* = 2.0 | 2´ |
| 6.89, 1 H, d, *J* = 8.2 | 5 |
| 7.08, 1 H, dd, *J* = 8.2, 2.1 Hz | 6 |
| 7.19, 1 H, d, *J* = 2.1 Hz | 2 |
| 7.59, 1 H, d, *J* = 15.9 Hz | 7 |

**
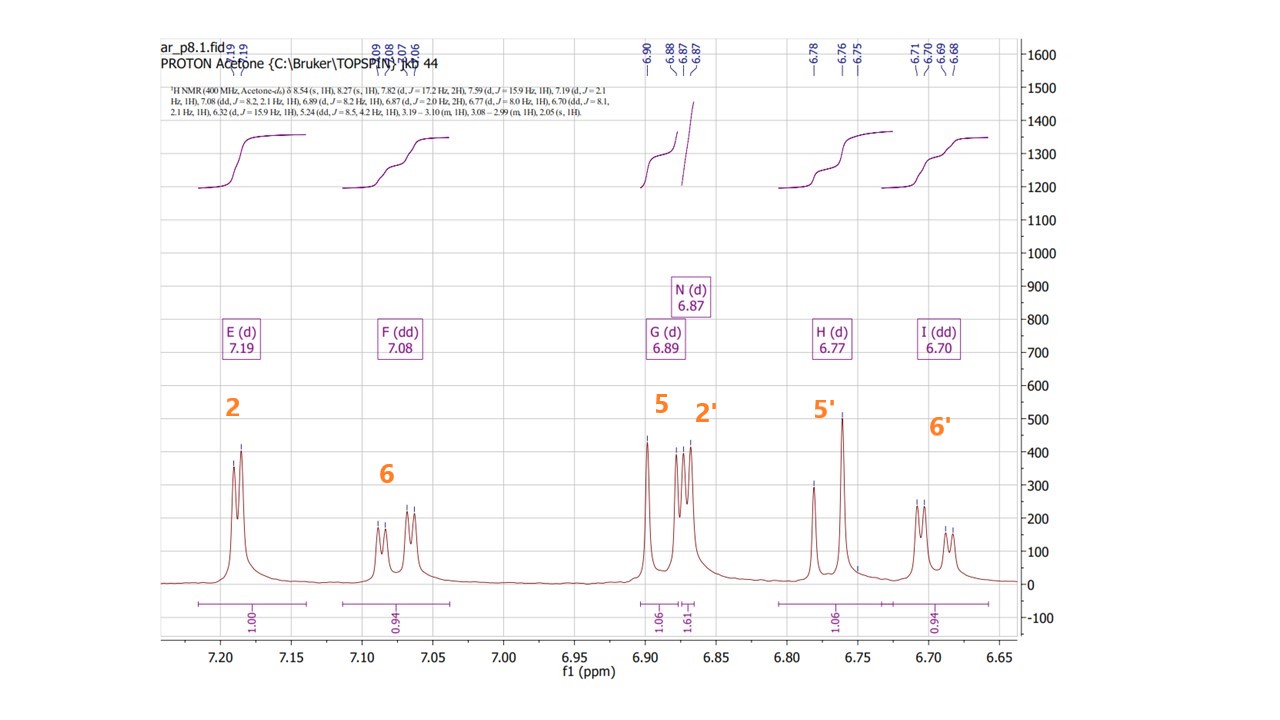
**

**S2. ^13^CNMR spectrum of rosmarinic acid (101 MHz acetone d_6_)**


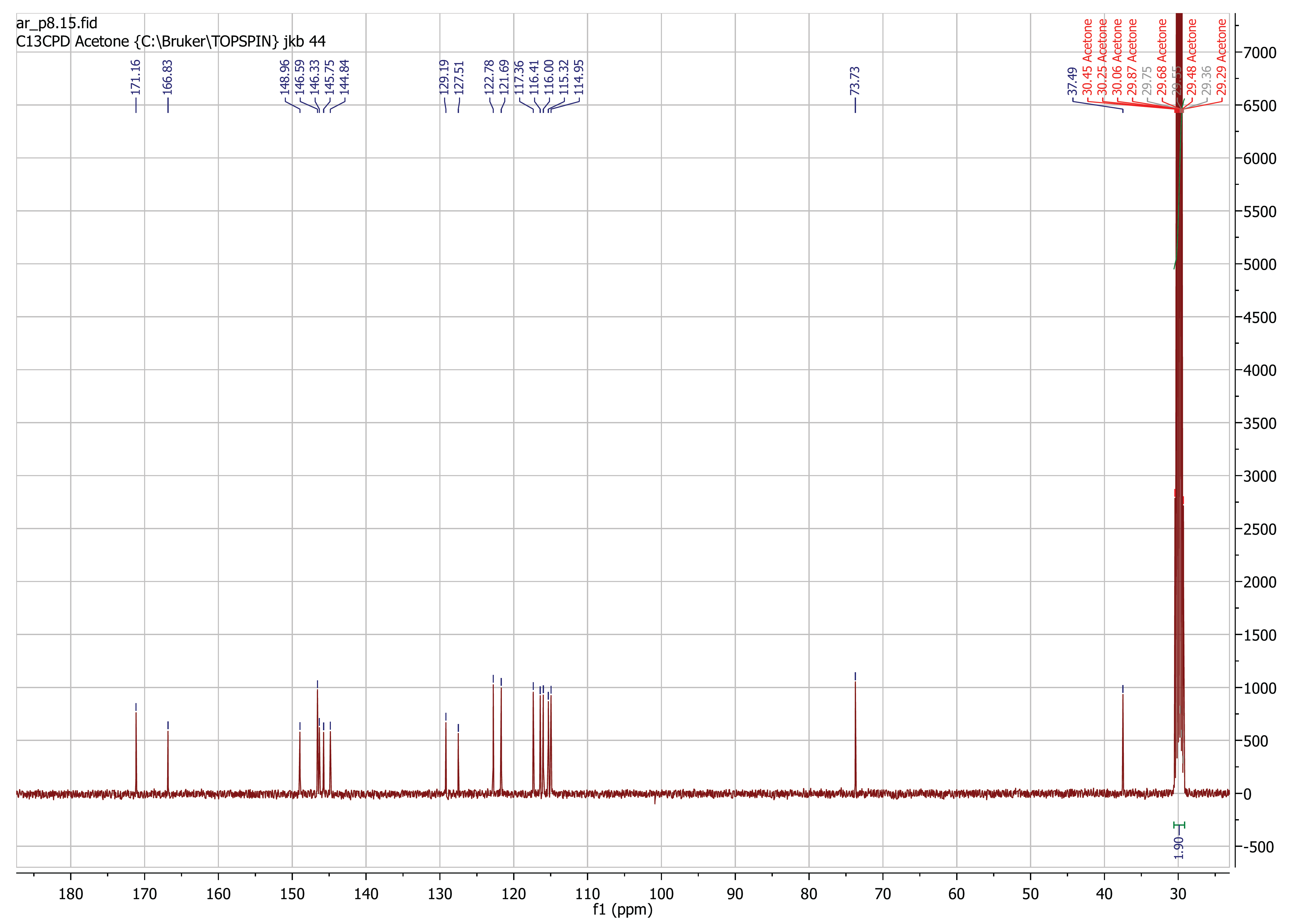

| Carbon | ppm | Wang et al. [**43]** |
| --- | --- | --- |
| 7’ | 37.49 | 37.4 |
| 8’ | 73.73 | 73.7 |
| 8 | 114.95 | 114.8 |
| 2 | 115.32 | 115.2 |
| 5’ | 116.00 | 115.5 |
| 5 | 116.41 | 116.3 |
| 2’ | 117.36 | 117.3 |
| 6’ | 121.69 | 121.6 |
| 6 | 122.78 | 122.7 |
| 1 | 127.51 | 127.3 |
| 1’ | 129.19 | 129.1 |
| 4’ | 144.84 | 144.7 |
| 3’ | 145.75 | 145.7 |
| 7 | 146.33 | 146.3 |
| 3 | 146.59 | 146.5 |
| 4 | 148.95 | 149.0 |
| 9 | 166.83 | 166.8 |
| 9’ | 171.16 | 171.2 |

**S3. References of supplementary material**

[1] Gao, Y., Sicker, D., Zeller, K., Siehl, H., Berger, S. *Rosmarinic acid* in Sicker, D., Zeller, K., Siehl, H., Berger, S. (Eds.) *Natural Products: Isolation, Structure Elucidation, History.*  391-410. (Wiley-VCH 2019).
